# SARS-CoV-2 non-structural protein 13 (nsp13) hijacks host deubiquitinase USP13 and counteracts host antiviral immune response

**DOI:** 10.1038/s41392-021-00509-3

**Published:** 2021-03-11

**Authors:** Guijie Guo, Ming Gao, Xiaochen Gao, Bibo Zhu, Jinzhou Huang, Kuntian Luo, Yong Zhang, Jie Sun, Min Deng, Zhenkun Lou

**Affiliations:** 1 Department of Molecular Pharmacology and Experimental Therapeutics, Mayo Clinic, Rochester, MN USA; 2 Department of Oncology, Mayo Clinic, Rochester, MN USA; 3 Thoracic Diseases Research Unit, Division of Pulmonary and Critical Care Medicine, Department of Medicine, Mayo Clinic College of Medicine and Science, Rochester, MN USA; 4 Department of Immunology, Mayo Clinic College of Medicine and Science, Rochester, MN USA

**Keywords:** Cell biology, Molecular biology

**Dear Editor**,

COVID-19 (Coronavirus Disease-2019), a respiratory disease caused by the novel virus strain, SARS-CoV-2 (severe acute respiratory syndrome coronavirus 2), an enveloped, positive-sense, single-stranded RNA betacoronavirus of the family *Coronaviridae*, has spread worldwide.^1^ Notably, SARS-CoV-2 infection led to poor induction of type I interferon response, and the impaired type I IFN responses have been shown to be associated with severe COVID-19 disease.^2^ However, molecular mechanisms by which SARS-CoV-2 suppresses type I IFN production, and how host cells respond to the inhibition of type I IFN response during SARS-CoV-2 infection, remain largely unknown.

Here, we found that SARS-CoV-2 non-structural protein 13 (nsp13) has an inhibitory role in regulating type I interferon production. nsp13 overexpression suppressed IFN-β levels induced by RNA virus mimics (3p-hpRNA, Poly: IC) and influenza virus (Fig. 1a–c and Supplementary Fig. S1). We next examined the interaction of nsp13 with core components of host antiviral immune signaling initiated by RNA virus, and found that nsp13 interacts with TBK1 but not others (Fig. 1d and Supplementary Fig. S2a, b), implying that nsp13 might suppress type I IFN production through interacting with TBK1. TBK1 has to be recruited to MAVS that serves as a scaffold to bring IRF3 and TBK1 into proximity, thereby facilitating IRF3 by TBK1.^3^ Thus, we asked whether nsp13 suppresses the recruitment of TBK1 to MAVS, and consequently disrupts TBK1-mediated IRF3 phosphorylation. As expected, the interaction of MAVS and TBK1 was impaired in the presence of nsp13 (Fig. 1e).

**Fig. 1:**
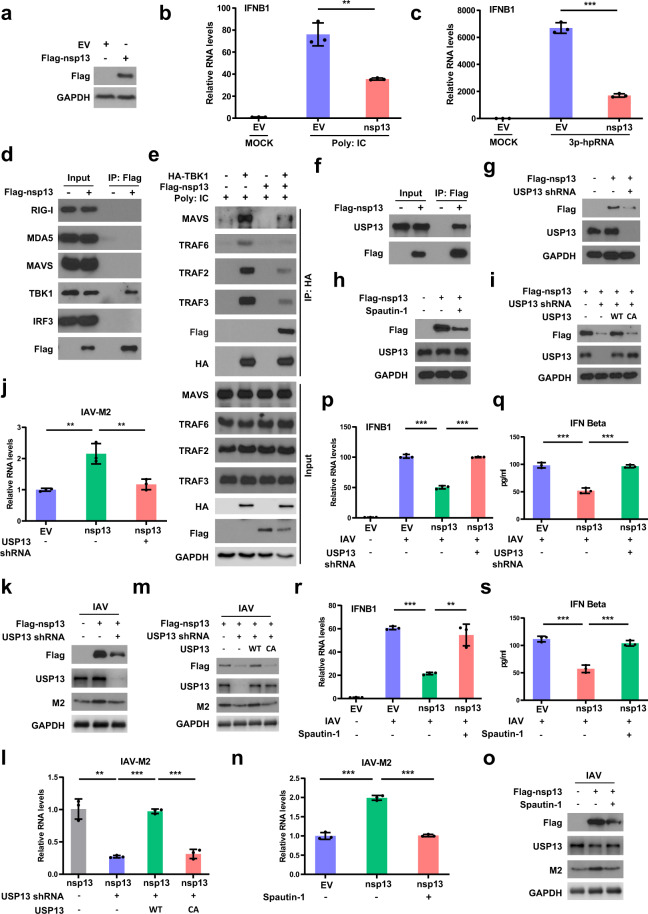
SARS-CoV-2 non-structural protein 13 (nsp13) hijacks host deubiquitinase USP13 to stabilize itself and counteracts host antiviral immune response. **a** HEK293T cells were transfected with empty vector (EV) or Flag-nsp13. The cells were then lysed and blotted with indicated antibodies. **b** The IFN-β RNA levels in control and nsp13 expressing cells transfected with Poly: IC for 8–12 h were analyzed by qRT-PCR. **c** Control and nsp13 expressing cells were transfected with 3p-hpRNA for 8–12 h, and then harvested to analyze the IFN-β RNA levels by qRT-PCR. **d** HEK293T cells were transfected with EV or Flag-nsp13. The cells were then lysed and immunoprecipitated with anti-Flag agarose beads. The beads were boiled and probed with indicated antibodies. **e** Control and nsp13 expressing cells were transfected with HA-TBK1 and treated with Poly: IC. The cells were then lysed and immunoprecipitated with anti-HA agarose beads. The beads were boiled and analyzed with indicated antibodies. **f** A549 cells were transfected with Flag-nsp13 followed by immunoprecipitation assay. The interaction of nsp13 and USP13 was detected by Western blot. **g** nsp13 expression in control or USP13 knockdown A549 cells was detected by Western blot. **h** Western blot analysis of nsp13 expression in A549 cells treated with spautin-1. **i** nsp13 expression in USP13 knockdown A549 cells re-expressing indicated constructs was detected by Western blot. **j**, **k** nsp13 expressing A549 cells stably expressing USP13 shRNA were infected with influenza virus A/PR/8/34. The M2 RNA (**j**) and protein (**k**) levels in cells were analyzed by qRT-PCR and Western blot, respectively. **l**, **m** A549 cells expressing indicated constructs were infected with influenza virus A/PR/8/34, and then harvested to detect the RNA (**l)** and protein (**m**) levels of M2 by qRT-PCR and immunoblotting, respectively. **n**–**o** Control and nsp13 expressing A549 cells were treated with spautin-1 and then infected with influenza virus A/PR/8/34. The M2 RNA (**n**) and protein (**o**) levels in cells were examined by qRT-PCR and Western blot, respectively. **p**, **q** nsp13 expressing A549 cells stably expressing USP13 shRNA were infected with influenza virus A/PR/8/34. The RNA (**p**) and protein (**q**) levels of IFN-β in cells were detected by qRT-PCR and ELISA, respectively. **r**, **s** Control and nsp13 expressing A549 cells treated with spautin-1 were infected with influenza virus A/PR/8/34. The IFN-β RNA (**r**) and protein (**s**) levels in cells were examined by qRT-PCR and ELISA, respectively. Data are shown as mean ± SEM from three independent experiments. *p* value was determined by two-tailed unpaired *t* test (*p < 0.05; **p < 0.01; ***p < 0.001)

TRAF family has been shown to be responsible for the recruitment of TBK1 to MAVS,^4^ thus we asked whether nsp13 affects the interaction of TBK1 and TRAFs. nsp13 overexpression impeded the interaction between TBK1 and TRAF2/3/6 (Fig. 1e). Consistently, we found that the scaffold dimerization domain (SDD) of TBK1 that is responsible for the TRAFs–TBK1 interaction, is required for the interaction of nsp13 and TBK1 (Supplementary Fig. S2c), suggesting that nsp13 competes with TRAFs to bind to TBK1, which could explain the reduced interaction of TBK1 and TRAFs in the presence of nsp13 (Fig. 1e). These results suggest that nsp13 suppresses type I IFN production by disrupting the association of TBK1 with MAVS.

Next, we studied how host cells respond to the inhibitory role of nsp13 in regulating type I IFN response. The ubiquitination and deubiquitination system play critical roles in regulating multiple signaling pathways, including protein degradation, interaction, and activation.^5^ We hypothesized that host cells may employ the ubiquitination system to target nsp13 for degradation and thereby defending against virus infection, while nsp13 in turn might hijack the host deubiquitination system to prevent itself from degradation. Therefore, we performed a screen assay to identify potential deubiquitinases (DUBs) that interact with nsp13, which might be explored for therapeutic use.

USP13 was identified to interact with nsp13 (Fig. 1f and Supplementary Fig. S3a, b). We examined nsp13 protein levels in USP13 knockdown cells, and found that nsp13 levels decreased in the absence of USP13, and the decrease of nsp13 levels could be reversed by the addition of proteasome inhibitor MG132 (Fig. 1g and Supplementary Fig. S3c, d), suggesting that USP13 regulates nsp13 levels in a proteasome-dependent manner. Notably, treatment with USP13 inhibitor spautin-1, which could block the deubiquitination activity of USP13, also led to the reduction of nsp13 levels (Fig. 1h and Supplementary Fig. S3e), suggesting that the enzymatic activity of USP13 may be required for its regulation of nsp13. Consistently, overexpression of wild type (WT), but not the catalytic-inactive (CA) mutant of USP13 with a mutation at the core enzymatic domain, could rescue the decreased nsp13 levels caused by USP13 depletion (Fig. 1i and Supplementary Fig. S3f), suggesting that USP13 can regulate nsp13 levels most likely by deubiquitinating and consequently stabilizing nsp13.

As expected, we found that USP13 regulates nsp13 ubiquitination in cells. Loss of USP13 led to the increase of ubiquitinated nsp13 (Supplementary Fig. S3g). Overexpression of WT, but not the CA mutant of USP13, decreased the ubiquitination of nsp13 (Supplementary Fig. S3h). These results suggest that nsp13 can hijack the host deubiquitinase USP13 to prevent itself from degradation, thereby suppressing type I IFN production and helping the virus to survive in host cells.

Next, we asked whether USP13 affects the inhibitory role of nsp13 in regulating type I IFN production, and whether USP13 inhibitor could be used to target nsp13 for degradation and thereby promoting type I IFN production and consequently suppressing virus replication in host cells. We knocked down USP13 in nsp13 expressing cells, and detected IFN-β levels. As shown in Supplementary Fig. S4a, nsp13 overexpression led to decrease of IFN-β levels, and loss of USP13 can reverse the decrease of IFN-β levels caused by nsp13 overexpression. Re-expression of WT, but not the CA mutant of USP13 in USP13-depleted cells, could restore the inhibition of IFN-β levels caused by nsp13 (Supplementary Fig. S4b). Intriguingly, treatment with USP13 inhibitor spautin-1 also reversed the decreased IFN-β levels caused by nsp13 overexpression (Supplementary Fig. S4c).

Above results prompted us to further evaluate the effects of USP13 and nsp13 on viral infection. Currently, we could not perform experiments using live SARS-CoV-2 in the lab. We thus used influenza virus as a model. This could also be relevant for potential interaction between SARS-CoV-2 and influenza virus infection. As shown in Fig. 1j, k and Supplementary Fig. S4d, much more viruses were detected in nsp13 overexpression cells than that in control cells. Depletion of USP13 led to the reduction of viruses caused by nsp13 overexpression. Additionally, the observed reduction of viruses caused by loss of USP13 in the presence of nsp13 can be reversed by overexpression of WT but not the CA mutant of USP13 (Fig. 1l, m and Supplementary Fig. S4e). Moreover, USP13 inhibitor spautin-1 treatment also led to significant decrease of viruses caused by nsp13 overexpression (Fig. 1n, o and Supplementary Fig. S4f). Consistently, dramatic decrease of IFN-β levels were observed in nsp13 overexpressing cells infected with influenza virus, which can be reversed by silence of USP13 or treatment with USP13 inhibitor (Fig. 1p–s and Supplementary Fig. S4g, h). Our results demonstrate that USP13 is hijacked to maintain nsp13 expression and the inhibitory role of nsp13 in regulating type I IFN production, and USP13 inhibitor could be employed to suppress virus replication by targeting nsp13 for degradation thereby disrupting its inhibitory role in regulating type I IFN production.

In summary, we demonstrated that SARS-CoV-2 nsp13 suppresses RNA virus-induced type I IFN production. Mechanistically, nsp13 interacts with TBK1, and the nsp13–TBK1 interaction impedes the association of TBK1 with TRAFs and consequent recruitment of TBK1 to MAVS, thereby suppressing TBK1-mediated IRF3 phosphorylation. In addition, we found that nsp13 takes advantage of host proteins to stabilize itself. We found that nsp13 interacts with the deubiquitinase USP13, which deubiquitinates and stabilizes nsp13. Loss of USP13 enhances ubiquitination of nsp13 and destabilizes nsp13 protein. Moreover, depletion of USP13 or treatment with USP13 inhibitor relieves the inhibitory role of nsp13 for type I IFN response and suppresses virus replication in host cells, suggesting that USP13 inhibitor could be employed to suppress virus replication by targeting nsp13 for degradation. Further studies are ongoing to investigate the anti-type I IFN activity of nsp13 in the circumstance of SARS-CoV-2 infection in vitro and in vivo, and to evaluate the efficiency of USP13 inhibitor in suppressing SARS-CoV-2 replication.

## Supplementary information

Supplementary Material

## Data Availability

Data are available upon reasonable request.
