# Supplementary material for: SARS-CoV-2 non-structural protein 13 (nsp13) hijacks host deubiquitinase USP13 and counteracts host antiviral immune response
Source: Signal Transduct Target Ther. 2021 Mar 11;6:119. doi: 10.1038/s41392-021-00509-3 (PMC7947159; doi:10.1038/s41392-021-00509-3)
Supplement: Supplementary file 1 — Supplementary Material [file 41392_2021_509_MOESM1_ESM.docx]

**Supplementary Materials for**

**SARS-CoV-2 non-structural protein 13 (nsp13) hijacks host deubiquitinase USP13 and counteracts host antiviral immune response**

Guijie Guo^1,2#^, Ming Gao^1,2#^, Xiaochen Gao^3,4^, Bibo Zhu^3,4^, Jinzhou Huang^1,2^, Kuntian Luo^1,2^, Yong Zhang^1,2^, Jie Sun^3,4^, Zhenkun Lou^1,2*^

^1^ Department of Molecular Pharmacology and Experimental Therapeutics, Mayo Clinic, Rochester, MN, 55905, USA

^2^ Department of Oncology, Mayo Clinic, Rochester, MN, 55905, USA

^3^ Thoracic Diseases Research Unit, Division of Pulmonary and Critical Care Medicine, Department of Medicine, Mayo Clinic College of Medicine and Science, Rochester, MN 55905, USA

^4^ Department of Immunology, Mayo Clinic College of Medicine and Science, Rochester, MN 55905, USA

^#^These authors contribute equally: Guijie Guo, Ming Gao

*Author for correspondence: [lou.zhenkun@mayo.edu](mailto:lou.zhenkun@mayo.edu)

**This PDF file includes:**

Materials and Methods

Supplementary Figures S1 to S4

**Materials and Methods**

**Cell culture**

HEK293T and A549 cell lines were purchased from ATCC. All cell lines have been tested and confirmed by the Mayo Clinic Medical Genome Facility. HEK293T and A549 were maintained in DMEM media with 10% FBS. Cells were kept in a humidified 37°C 5% CO2 incubator.

**Virus infection**

Influenza virus strain A/PR/8/34 (H1N1) was prepared as previously described.[^1^](#_ENREF_1) For infection, cells were washed with phosphate-buffered saline (PBS) and infected with A/PR/8/34 (H1N1). After 1 h adsorption, cells were washed once with warm phosphate-buffered saline (PBS) and then cultured with DMEM containing 1% FBS for indicated time.

**Plasmids, reagents** **and antibodies**

pCAG-nsp13-Flag was kindly provided by Pei-Hui Wang (Advanced Medical Research Institute, Shandong University). HA-USP13 was purchased from Addgene. USP13 site mutants were generated by site-directed mutagenesis (Stratagene). His-tagged ubiquitin was obtained from Addgene and has been previously described.[^2^](#_ENREF_2) FLAG agarose beads (A2220) were purchased from Sigma Aldrich. 3p-hpRNA (tlrl-hprna), and Poly: IC (tlrl-pic) were purchased from Invivogen. Human IFN-Beta ELISA Kit was purchased from PBL Assay Science. USP13 (GTX118595, dilution: 1:500) and Influenza A virus M2 (GTX125951, 1:1000) antibodies were purchased from Genetex. TBK1 (3504, 1:1000), TRAF6 (8028, 1:1000), TRAF2 (4724, 1:1000), TRAF3 (4729, 1:1000), RIG-I (3743, 1:1000), MDA5 (5321, 1:1000), MAVS (3993, 1:1000), IRF3 (4302, 1:1000), phospho-IRF3 (4947, 1:1000) antibodies were purchased from Cell Signaling Technology. Ub antibody (sc-8017, 1:2000) was purchased from Santa Cruz. HA (H9658, 1:1000) and FLAG (F3165, 1:1000) antibodies were purchased from Sigma. GAPDH (60004-1-Ig, 1:2000) antibody was purchased from Proteintech.

**RNA interference**

The following shRNAs from Sigma were used in this study:

USP13 shRNA-1: 5’-GCCAGTATCTAAATATGCCAA-3’,

USP13 shRNA-2: 5’-CCGGTGAAATCTGAACTCATT-3’,

USP13 shRNA-3: 5’-CGATTTAAATAGCGACGATTA-3’.

**Quantitative reverse transcription (RT)-PCR**

RNA was isolated with TRIzol RNA Isolation Reagents (Thermo Fisher). Reverse transcription was performed with PrimeScript™ RT Reagent Kit (Takara), and quantitative PCR was performed with Power SYBR Green Master Mix (Thermo Fisher). The primer sequences are available upon request. For quantification, the 2^-ΔΔCt^ method was used to calculate the relative RNA levels against GAPDH.

**Western blot and immunoprecipitation**

Cells were harvested and lysed with NETN buffer (20 mM Tris-HCl, pH 8.0, 100 mM NaCl, 1 mM EDTA, 0.5% Nonidet P-40 with 10 mM NaF, and 1 mg per ml each of pepstatin A and aprotinin). After centrifugation at 12000 x g for 15 min, supernatant containing proteins were immunoprecipitated by incubating indicated antibodies or agarose beads overnight at 4 °C. The immunoprecipitates were washed with NETN buffer, centrifuged at 800 x g for 1 min for three times. The immunoprecipitates were suspended with 50 μL 1x Laemmli buffer and boiled for SDS-PAGE. Immunoblotting was performed following standard procedures as previous described.[^3^](#_ENREF_3)

**Denaturing Ni-NTA pull-down**

Cells were harvested and lysed in Urea buffer (8 M Urea, 0.1 M NaH_2_PO4, 0.01 M Tris-HCl pH 8.0, and 300 mM NaCl). Lysates were then sonicated and incubated with Ni-NTA beads for 2 h at room temperature. After washing the beads with Urea wash buffer (8 M Urea, 0.1 M NaH_2_PO4, 0.01 M Tris-HCl pH 8.0, and 300 mM NaCl) for 5 times, the immunocomplexes were suspended with 1x Laemmli buffer and subjected to SDS-PAGE and immunoblotting.

**Statistical analysis**

Data in bar and line graphs are presented as mean ± S.E.M of three independent experiments. Statistical analyses were performed with the Student's *t*-test. Statistical significance is represented in figures by: *, *p*<0.05; **, *p*<0.01; ***, *p*<0.001.

**References**

1 Zhu, B. *et al.* BCL6 modulates tissue neutrophil survival and exacerbates pulmonary inflammation following influenza virus infection. *Proceedings of the National Academy of Sciences of the United States of America* **116**, 11888-11893 (2019).

2 Deng, M. *et al.* Deubiquitination and Activation of AMPK by USP10. *Molecular cell* **61**, 614-624 (2016).

3 Li, Y. *et al.* USP13 regulates the RAP80-BRCA1 complex dependent DNA damage response. *Nature communications* **8**, 15752 (2017).

**Fig. S1**


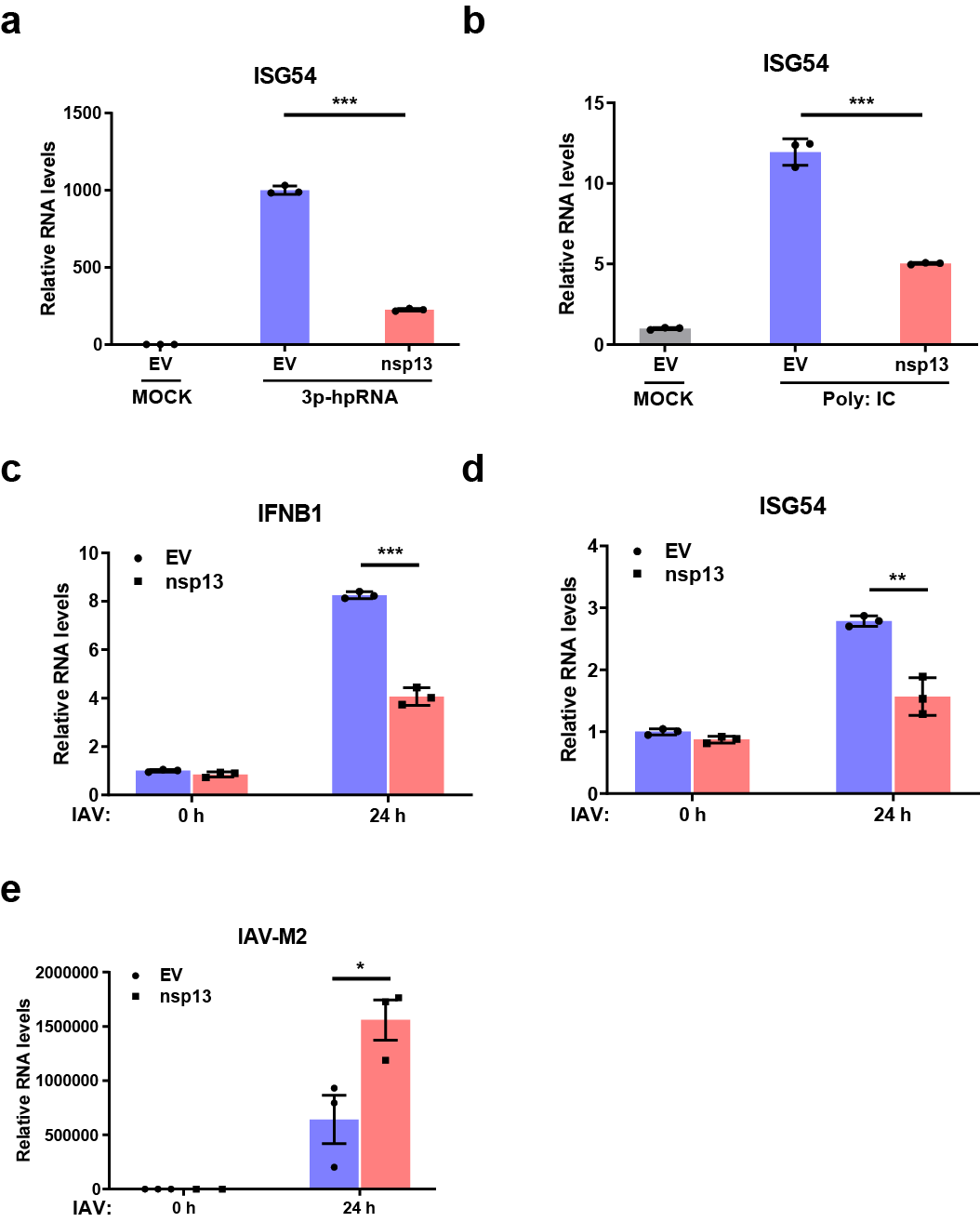


**Fig. S1 SARS-CoV-2 nsp13 suppresses RNA virus-induced IFN-β and ISG54 expression. a-b** The RNA levels of ISG54 in control and nsp13 expressing cells transfected with 3p-hpRNA **(a)**, Poly: IC **(b)**, were detected by qRT-PCR. **c** The IFN-β RNA levels in control and nsp13 expressing cells infected with influenza virus A/PR/8/34, were detected by qRT-PCR. **d** The RNA levels of ISG54 in control and nsp13 expressing cells infected with influenza virus A/PR/8/34, were detected by qRT-PCR. **e** qRT-PCR analysis of the M2 RNA levels in control and nsp13 expressing cells infected with influenza virus A/PR/8/34. Data are shown as mean ± SEM from three independent experiments. *p* value was determined by two-tailed unpaired t test (*, *p*<0.05; **, *p*<0.01; ***, *p*<0.001).

**Fig. S2**


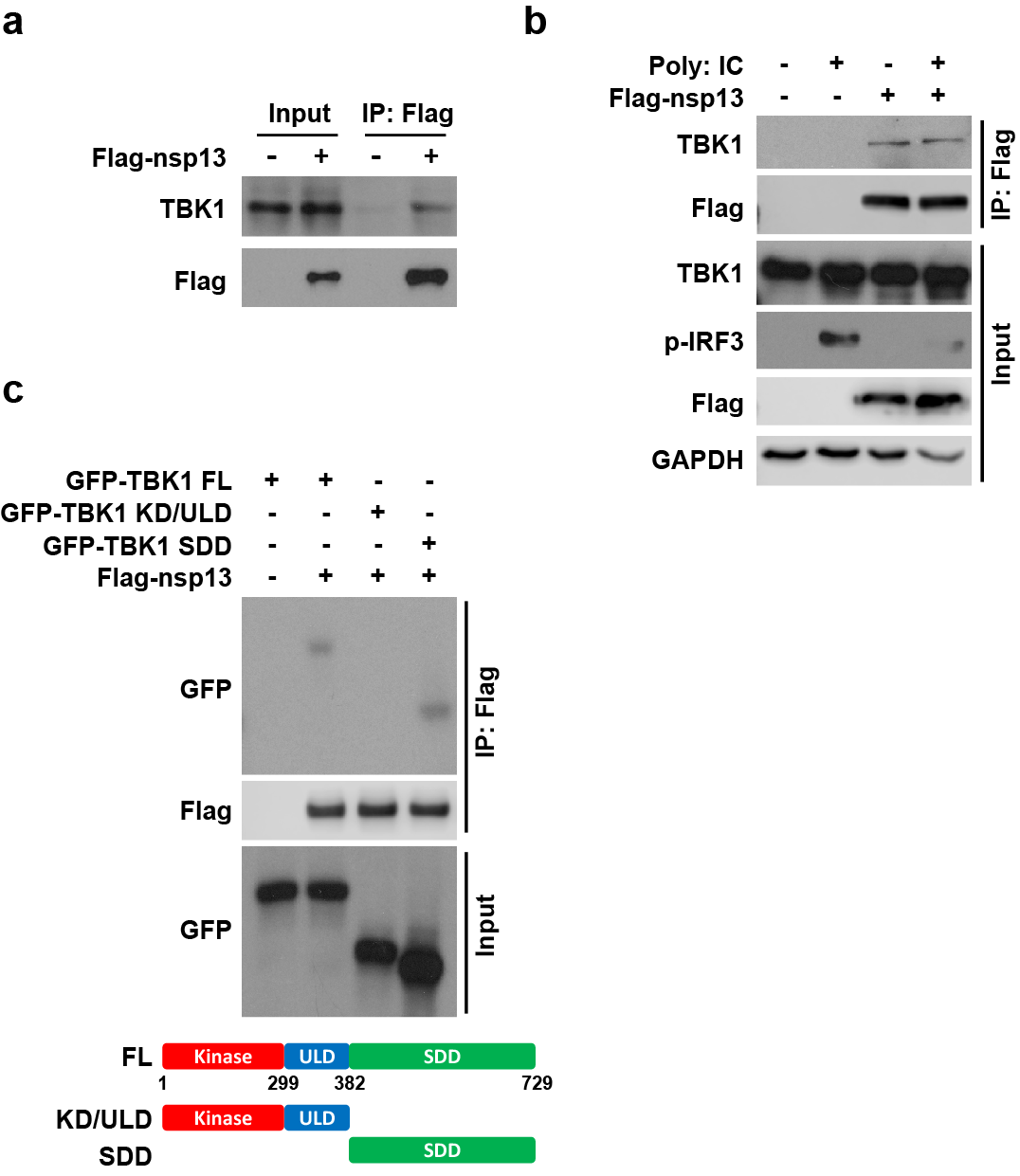


**Fig. S2 nsp13 interacts with TBK1. a** A549 cells were transfected with Flag-nsp13 followed by immunoprecipitation assay. The interaction of nsp13 and TBK1 was detected by Western blot as indicated. **b** HEK293T cells were transfected with empty vector or Flag-nsp13 and treated with Poly: IC. The cells were then lysed and immunoprecipitated with anti-Flag agarose beads. The beads were boiled and blotted with indicated antibodies. **c** Schematic representation of TBK1 constructs used in this study (bottom). 293T cells were transfected with indicated TBK1 constructs. Cell lysates were incubated with anti-Flag agarose beads. The immunoprecipitates were blotted with indicated antibodies (top).

**Fig. S3**


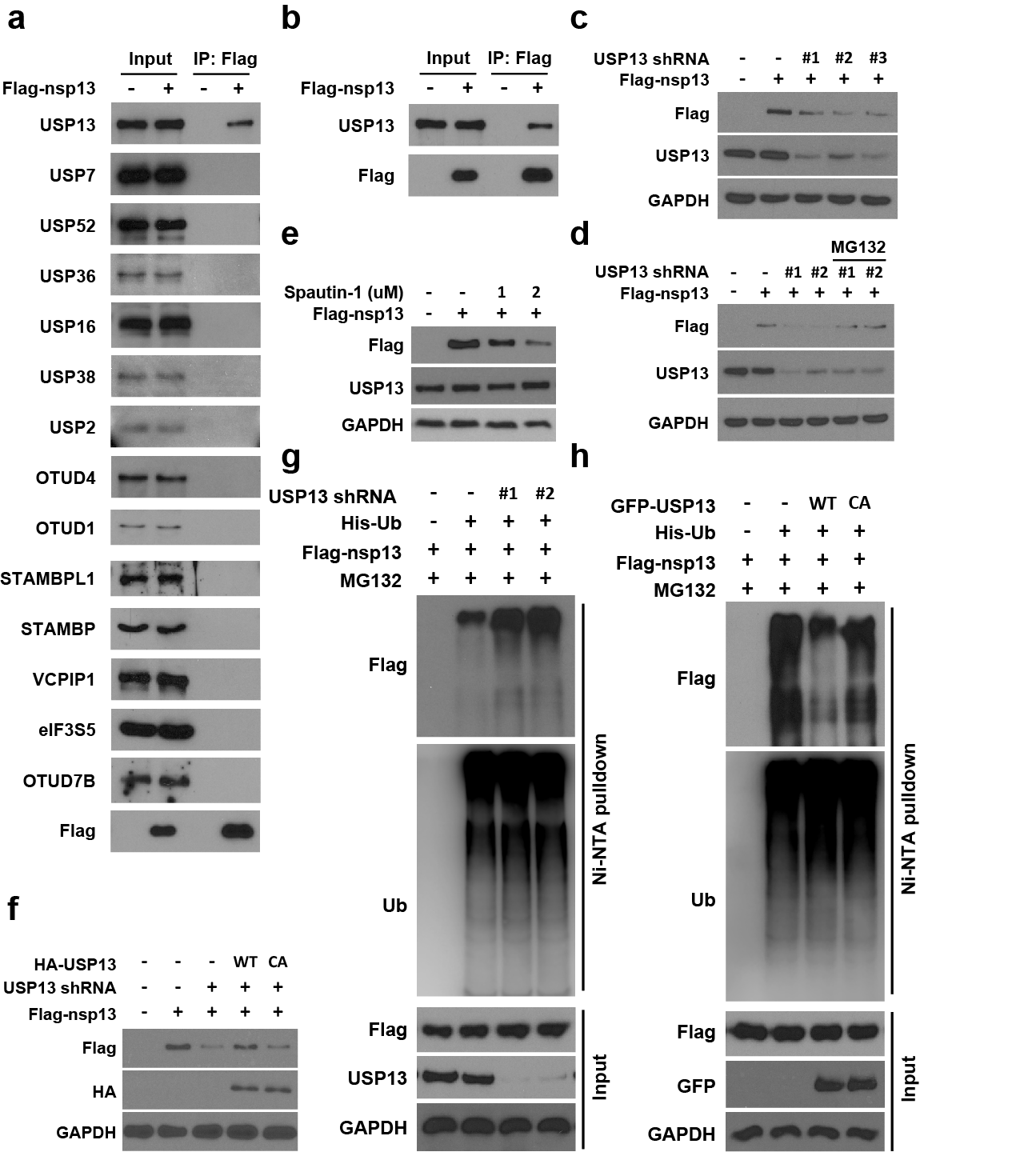


**Fig. S3 USP13 interacts with, deubiquitinates and stabilizes nsp13. a** HEK293T cells were transfected with Flag-nsp13 followed by immunoprecipitation assay. The interaction of nsp13 and a panel of deubiquitinases (DUBs) were detected by Western blot with indicated antibodies. **b** 293T cells were transfected with Flag-nsp13 followed by immunoprecipitation assay. The interaction of nsp13 and USP13 was detected by Western blot. **c** nsp13 protein levels in control and USP13 knockdown 293T cells were detected by Western blot. **d** nsp13 protein levels in control and USP13 knockdown 293T cells untreated or treated with MG132, were detected by immunoblotting. **e** nsp13 expressing HEK293T cells were treated with spautin-1 for 24 h, then lysed, subjected to SDS-PAGE, and probed with indicated antibodies. **f** nsp13 protein levels in control and USP13 knockdown cells reconstituted with indicated constructs were examined. **g** Control and USP13 knockdown cells were transfected with Flag-nsp13 and His-Ub. Cells were then harvested following treatment with MG132, lysed and immunoprecipitated using Ni-NTA beads. The lysates were boiled and probed with indicated antibodies. **h** Cells expressing indicated constructs were harvested following MG132 treatment, lysed and immunoprecipitated with Ni-NTA beads. Blots were detected by indicated antibodies.

**Fig. S4**


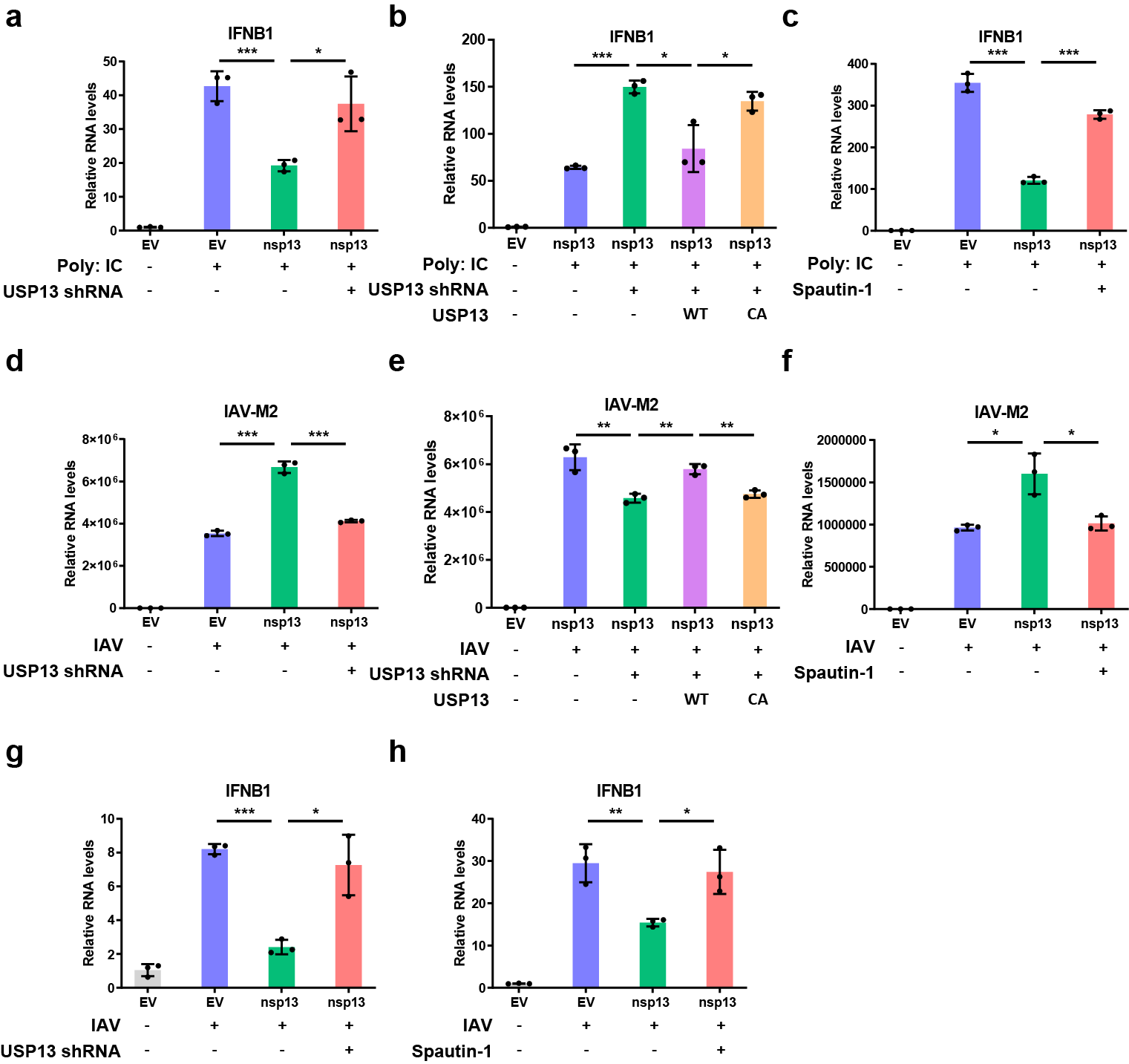


**Fig. S4 USP13 inhibitor suppresses virus replication by disrupting the inhibitory role of nsp13 in regulating Type I IFN production. a** nsp13 expressing HEK293T cells stably expressing USP13 shRNA were transfected with Poly: IC. The RNA levels of IFN-β were analyzed by qRT-PCR. **b** Cells expressing indicated constructs were transfected with Poly: IC, and then harvested to detect IFN-β RNA levels by qRT-PCR. **c** Control and nsp13 expressing cells were treated with spautin-1 and then transfected with Poly: IC. The RNA levels of IFN-β were examined by qRT-PCR. **d** nsp13 expressing HEK293T cells stably expressing USP13 shRNA were infected with influenza virus A/PR/8/34. The M2 RNA levels in cells were analyzed by qRT-PCR. **e** Cells expressing indicated constructs were infected with influenza virus A/PR/8/34, and then harvested to detect the M2 RNA levels by qRT-PCR. **f** Control and nsp13 expressing cells were treated with spautin-1 and then infected with influenza virus A/PR/8/34. The M2 RNA levels in cells were examined by qRT-PCR. **g** nsp13 expressing cells stably expressing USP13 shRNA were infected with influenza virus A/PR/8/34. The RNA levels of IFN-β in cells were detected by qRT-PCR. **h** Control and nsp13 expressing cells treated with spautin-1 were infected with influenza virus A/PR/8/34. The IFN-β RNA levels in cells were examined by qRT-PCR. Data are shown as mean ± SEM from three independent experiments. *p* value was determined by two-tailed unpaired t test (*, *p*<0.05; **, *p*<0.01; ***, *p*<0.001).
